# Mesoporous Silica Nanoparticles as Theranostic Antitumoral Nanomedicines

**DOI:** 10.3390/pharmaceutics12100957

**Published:** 2020-10-11

**Authors:** Alejandro Baeza, Maria Vallet-Regí

**Affiliations:** 1 Dpto. Materiales y Producción Aeroespacial, ETSI Aeronáutica y del Espacio, Universidad Politécnica de Madrid, 28040 Madrid, Spain; 2 Dpto. Química en Ciencias Farmacéuticas, Instituto de Investigación Sanitaria, Universidad Complutense de Madrid, Hospital 12 de Octubre i+12, Plaza Ramón y Cajal s/n, CIBER de Bioingeniería, Biomateriales y Nanomedicina, CIBER-BBN, 28040 Madrid, Spain

**Keywords:** nanomedicine, mesoporous silica nanoparticles, theranostics, antitumoral therapy

## Abstract

Nanoparticles have become a powerful tool in oncology not only as carrier of the highly toxic chemotherapeutic drugs but also as imaging contrast agents that provide valuable information about the state of the disease and its progression. The enhanced permeation and retention effect for loaded nanocarriers in tumors allow substantial improvement of selectivity and safety of anticancer nanomedicines. Additionally, the possibility to design stimuli-responsive nanocarriers able to release their payload in response to specific stimuli provide an excellent control on the administered dosage. The aim of this review is not to present a comprehensive revision of the different theranostic mesoporous silica nanoparticles (MSN) which have been published in the recent years but just to describe a few selected examples to offer a panoramic view to the reader about the suitability and effectiveness of these nanocarriers in the oncology field.

## 1. Introduction

Nanoparticles have become a powerful tool in the clinical field, not only by their capacity to deliver drugs to diseased tissues in a controlled and selective manner [1], but also by their ability to simultaneously provide information about the stage of the disease progression [2]. Nanosystems endowed with these dual functions, drug delivery, and diagnosis, received the name of theranostic nanocarriers. Among the different clinical applications of these smart nanocarriers, antitumoral therapy have received the highest attention [3]. The origin of this interest began in 1986, when Maeda and Matsumura reported the passive tendency of intravenously injected nanometric agents to be accumulated within solid tumors [4]. The accelerated growth of solid tumors demands the rapid construction of tumoral blood vessels in order to provide the required nutrients to sustain them. These vessels are built in an uncontrollable manner presenting wide interendothelial junctions which create pores with diameters with up to a few hundreds of nanometers [5]. Thus, when nanoparticles that were injected in the blood stream reach the tumoral tissue, they can pass through these fenestrations being selectively accumulated there. Moreover, solid tumors usually present inefficient drainage system. This is a consequence of the compression of lymphatic vessels due the expansion of tumoral tissue, which enhances the retention time of the nanoparticles within the diseased tissue. The combination of these two characteristics, high permeability of tumoral blood vessels and inefficient drainage, constitutes to the gold standard of the use of nanoparticles in oncology and receives the name of enhanced permeation and retention (EPR) effect [6]. The discovery of EPR effect triggered the race to design nanocarriers capable to selectively deliver chemotherapeutic agents to tumoral tissue reducing the systemic toxicity caused by these drugs in healthy tissues. Thus, a vast number of different nanoparticles composed of both organic materials as polymers [7], lipids [8], and dendrimers [9] as well as by inorganic ones such as metals [10], ceramics [11], and carbon allotropes [12], among others have been reported exhibiting excellent drug delivery capacities. The selectivity of these nanocarriers has been enhanced anchoring targeting moieties such as antibodies [13], aptamers [14], proteins [15], peptides [16], and small molecules [17] on their surface. Additionally, the incorporation within the nanocarrier of imaging agents as molecular fluorophores [18], magnetic nanocrystals [19], ultrasound contrast agents [20], or radionuclides [21] allow the combination of therapy and diagnosis in one single theranostic nanocarrier. In the recent years, EPR effect has been object of an intense debate by the scientific community because many human tumors lack these highly permeable blood vessels and therefore, the efficacy of nanomedicines could be compromised [22]. Wilhem et al. have reported that only around 0.7% of the injected nanoparticles are accumulated in tumoral tissues that it would be not enough to induce significant therapeutic effects [23]. However, some recent studies suggested that nanoparticles enter into solid tumors by active mechanisms of transcytosis instead of by passive diffusion through the tumoral blood vessel gaps [24,25]. Moreover, increasing the dose of nanoparticles through co-administration of drug-loaded and empty nanocarriers improves nanoparticle accumulation in tumors up to 12% by saturation of Kupffer cells which are one of the main players of nanoparticle clearance [26]. Additionally, several other barriers as interstitial pressure within the tumor, hemodynamic considerations, or off target cellular uptake should be addressed to improve the selectivity and efficacy of nanomedicines [27,28]. Among the high variety of nanomaterials capable of transporting drugs, each of them with their advantages and liabilities, mesoporous silica nanoparticles (MSN) stands out by their unique properties as high loading capacity because of their external surface in the range of 1000 m^2^ per gram, high chemical, mechanical and thermal robustness, easy functionalization with different moieties and excellent biocompatibility [29]. MSN present a honeycomb-like pore network in which the pore channels are parallel without any interconnections between them. This unique pore network allows an outstanding control of the drug release behavior because of the capacity to block the pore outlets with different gatekeepers sensible to external or internal stimuli [30,31]. Thus, different gatekeepers can be anchored on the pore outlets through stimuli-responsive cross-linkers or responsive polymer shells in order to control the drug release process avoiding or allowing the drug departure in response to specific internal (pH, redox, enzymes, etc.,) or external stimulus (temperature, magnetic fields, ultrasounds, light) [32]. Additionally, MSN surface can be easily decorated with different functionalities as amino, thiol, or carboxylic groups, among others, which enhance the affinity of the housed drugs by the pore walls improving the drug-loading capacity [33]. There are different synthetic methods to produce MSN with multiple sizes, shapes, pore diameter, and pore network structures [34]. This variety allows to produce MSN designed to house diverse therapeutic agents, from small cytotoxic drugs to large proteins or oligonucleotide strands. These smart MSN are an elegant platform to deliver therapeutic agents to tumoral tissues enhancing not only the selectivity in the delivery but also controlling the administered dosage with high accuracy. The aim of this review is not to present a comprehensive revision of the different theranostic MSN which have been published in the recent years [35,36] but just to describe a few selected examples to offer a panoramic view to the reader about the suitability and effectiveness of these nanocarriers in the oncology field. For the sake of clarity, these nanosystems are categorized in different sections according to the associated imaging technique.

## 2. Optical Theranostics

**Figure 1:**
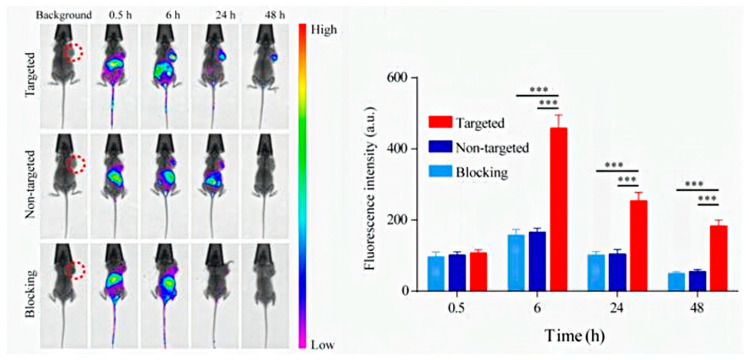
In vivo imaging of tumor-bearing mice after the injection of mesoporous silica nanoparticles (MSN) decorated with YQ26 aptamer. Left image shows the accumulation of nanocarriers in the tumor located in the flank of the mice. The graph located in the right quantified the accumulation of nanocarrier by fluorescence. ∗∗∗ *p* < 0.001. Reproduced from [41], Ivyspring International Publisher, 2017.

Light has been widely employed in nanomedicine both to trigger drug release and to interrogate the diseased tissue in order to harvest clinical information [37]. This stimulus present advantages such as excellent biocompatibility and easiness to focalize its action in specific zones. However, it also exhibits a strong limitation which is its poor penetration in biological tissues. Light in the visible (VIS) and ultraviolet region (UV) are rapidly adsorbed by hemoglobin and therefore, it barely penetrates in the tissue. Near infrared radiation (NIR) is scarcely adsorbed and can penetrate up to 10 cm into biological tissues. This light source is being widely used to treat tumoral tissues by photodynamic therapy (PDT). PDT is based on the use of specific molecules, called photosensitizers, capable to generate singlet oxygen species under exposition of NIR. One of the main problem of PDT is the non-selective biodistribution of the administered photosensitizers which can cause side reactions as skin photosensitivity. In order to avoid this problem, photosensitizers have been loaded in targeted nanoparticles which can be selectively located in the diseased tissue. Hollow MSN have been loaded with indocyanine green (ICG) which is a FDA approved photosensitizer that exhibit a short blood half-life of 2–4 min if it is intravenously administered in free form [38]. When ICG is loaded within the nanoparticle, its capacity to generate singlet oxygen species is reduced because of self-quenching caused by aggregation. Therefore, this photosensitizer is transported in an inactivated (OFF) state. Once the nanocarriers are engulfed by tumoral cells, ICG is released inside the cell being activated (ON) to generate oxidative species under NIR capable to destroy the tumoral cells. Zhang et al. have decorated the external surface of MSN with metalloproteinase-sensitive fluorophores which are only activated in the tumoral tissue [39]. Certain proteolytic enzymes as metalloproteinases MMP-2 are overproduced by tumoral tissues to digest the extracellular matrix (ECM) allowing the colonization of the surrounding tissues by malignant cells. TAMRA fluorophore was conjugated to a specific peptide Pro-Leu-Gly-Val-Arg (PLGVR) which is cleavable by MMP-2. On the other end of this peptide, a fluorescence quencher (Dabcyl) was also anchored to suppress the TAMRA fluorescence. These nanocarriers were loaded with a potent cytotoxic drug, camptothecin (CPT), which was retained inside the pore network by the presence of these moieties on pore outlets that act as pore blockers. When the nanoparticle reaches the tumoral tissue, the presence of MMP-2 cleaves this peptide triggering the drug departure and activating the TAMRA fluorescence that becomes visible to the nanoparticle by fluorescence imaging. The selectivity against tumoral cells was provided by anchoring cyclo-RGD which binds to αβ-integrins receptors usually overexpressed by many tumoral cell lines. Jeon et al. have encapsulated 1,1′-dioctadecyl-3,3,30,30-tetrametylindotrucarbocyanine iodide (DIR) within the pore network of MSN decorated with polyethyleneglycol (PEG) [40]. The presence of this dye allows the visualization of these nanoparticles by in vivo fluorescence imaging which have been used to monitor tumoral tissues and ischemic lesions. Additionally, laser irradiation at 808 nm of breast tumors in mice model treated with these DIR-loaded nanocarriers showed a significant tumor reduction by photothermal therapy (PTT) due to temperature increase in the zone. Fluorescent MSN decorated with the aptamer YQ26 have been employed to visualize tumoral blood vessels in mice models [41]. Aptamers are single-stranded DNA or RNA sequences that adopt 3D conformations able to selectively bind to certain molecules as proteins, peptides, sugars, ions, or phospholipids. The specific binding capacity of these oligonucleotide strands have been used to enhance the selectivity of the nanomedicines. YQ26 shows excellent capacity to bind to the homodimeric transmembrane glycoprotein, endoglin (END), which is highly expressed by tumoral blood vessels but not by healthy ones. The injection of these fluorescent nanoparticles allowed the in vivo visualization of the tumoral vasculature (Figure 1).

Yao et al. have developed MSN capped with cell-penetrating polydisulfides. These pore-blockers enhance cell endocytosis at the same time that maintain the drug housed within the nanocarrier until they are degraded by reductive species as glutathione present in the intracellular space [42]. These nanocarriers were capable of releasing small molecule inhibitors and anti-miR-21. The release of these agents caused tumoral cell apoptosis. Additionally, the incorporation of caspase-sensitive fluorogenic probe into the MSN surface provided information of its own apoptotic process. Apoptosis is a cell death program mediated by the expression of certain proteolytic enzymes known as caspases. Thus, the apparition of fluorescence inside tumoral cells treated with these nanocarriers confirm the apoptosis activation induced by the release of the transported drugs. The incorporation of molecular probes sensible to light is one way of providing optical properties to the nanocarriers, but the other strategy is to incorporate other nanoparticles within a nanocarrier. Carbon dots (CDs) are excellent optical probes because of their high photostability, low toxicity, and water dispersibility. Hollow MSN that contains CDs and doxorubicin were intratumorally injected into breast tumor mice models inducing a potent tumoral growth inhibition which was observed by in vivo fluorescent imaging [43]. Dox release was accelerated by the more acidic conditions present in the tumoral tissue and also in the endosomes of the tumoral cells. CD have been anchored on the pore outlets through redox-sensitive linkers in order to block drug departure and to act as fluorescent imaging agents at the same time [44]. This design allows a better control on drug release because it only takes place when the nanocarrier are exposed to the reductive environment present in the cytosol. The size and shape of CD determine the optical window in which they can be observed which enhances their potential applications [45]. CuS nanoparticles have been incorporated on the surface of MSN to exploit their capacity to generate mild hyperthermia in the tumoral tissue by exposition to NIR [46]. MSN surface was decorated with thiol groups to provide anchoring points to CuS nanoparticles. Dox was loaded within the pore network, with their release being mediated by the presence of three stimuli, glutathione within the tumoral cells, mild acidic conditions in the tumor, and laser irradiation. Dox release was monitored by the red fluorescence signal of this molecule.

## 3. Photoacustic Theranostics

An imaging technique that combines light and ultrasound is photoacoustic imaging (PA). PA employs light contrast agents that produce heat under NIR irradiation. This heat induces thermoelastic changes in the tissue which generate ultrasound waves that can be detected by US transducers. One widely photoacoustic contrast agent is ICG. This dye has been covalently grafted on MSN which contain on the surface a chemotherapeutic drug, mitoxantrone (MTX), which also exhibits NIR adsorption properties [47]. The conjugation of ICG on MSN increases the photoluminescence efficiency as well as its chemical stability. Injection of these nanocarriers in syngeneic breast tumor murine model showed that both PA signals corresponding to ICG and MTX were detected at 810 nm and 700 nm, respectively. Furthermore, the evaluation of the ratio PA_700 nm_/PA_810 nm_ confirmed the MTX release due to the significant reduction of MTX signal during the time. ICG has been encapsulated in core-shell MSN which contains a upconversion nanoparticle core formed by two different composition of NaYF4/Yb/Er [48]. Irradiation of these nanocarriers at 800 nm induced light harvesting of NIR and upconversion to green and red luminescence by the core which can be employed for optical imaging and heat production by ICG that can be detected by PA. Nanocarriers injected in brain vessels of mice were visualized by PA during longer times than free ICG as a consequence of the longer stability and residence time in the tissue. Finally, the PTT efficacy of these nanoparticles was tested in tumor-bearing mice models by intratumoral injection followed by NIR irradiation after one week. Mice treated with the nanocarriers and exposed to NIR exhibited smaller tumor sizes and growth in comparison with non-treated animals. ICG has been encapsulated in liposomes which wrapped Gd-doped MSN loaded with Dox to combine therapy, by light-responsive Dox release induced by the heat generated under NIR that distort the liposomal coating, and imaging with PA, fluorescence imaging and MRI [49]. Perfluoropentane (PFP) have been loaded within core-shell nanoparticles formed by a core of gold nanorods and a mesoporous silica shell [50]. When the system was irradiated with NIR at 808 nm, the gold nanorods generated enough heat to induce liquid–gas phase transition of PFP. This process produced PFP bubbles which were transformed into microbubbles by coalescence. The generation of these microbubbles enhanced the accumulation of the nanocarriers within the malignant tissue [50]. These nanoparticles were detected by PA and ultrasound (US) imaging because of the echogenic properties of the generated microbubbles. The efficacy of the system was evaluated in melanoma-bearing mice model in which the point of maximal nanoparticle accumulation in the tumor (24 h) was determined by PA and US imaging. At this time, the tumors were irradiated with NIR laser at 808 nm (1 W·cm^−2^ during 5 min) reaching a temperature in the tissue of 46.2 °C. Tumors treated with these nanocarriers and NIR exposition were completely eliminated on day 18 after the injection.

## 4. Ultrasound Theranostics

**Figure 2:**
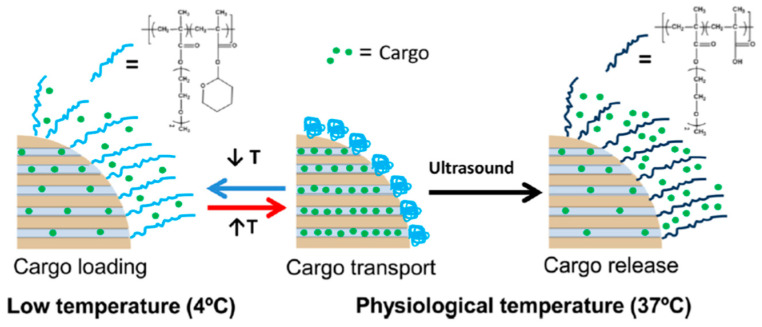
Ultrasound (US)-sensitive polymer coating to control drug-departure in MSN. Polymer shell collapsed at physiological temperature blocking drug departure while adopting an extended conformation under US releasing the payload. Reproduced with permission from [57], American Chemical Society, 2015.

US imaging is one of the most employed technique in the clinical practice because of its high penetration capacity, low cost, non-invasive, and non-ionizing nature. Different gas-filled organic nanocarriers have been employed to generate micro-bubbles acting as echogenic nanomaterials [51]. The micro-bubbles encapsulated in elastic shells vibrate and oscillate under US application acting as contrast agents. However, these nanocarriers usually present large sizes in the micrometer scale and low stability that compromise their clinical applications. Inorganic US contrast agents have higher stability and can be fabricated with smaller sizes than their organic counterparts [52]. In this case, the contrast agent properties of these rigid systems are based on the inhomogeneity that they introduce in the tissue instead of vibrations. Different inorganic nanomaterials have been employed as US contrast agents such as Au, TiO_2_, carbon nanotubes, and MSN [53]. MSN functionalized with the monoclonal antibody Herceptin^®^ have been employed to treat and diagnose breast cancer [54]. Herceptin^®^ binds to the human epidermal growth factor receptor 2 (HER2), which is usually upregulated in some types of breast tumors. In vitro culture of HER2 + breast cancer cells treated with anti-Her2-MSN produced a spike in the US contrast around 640% with a boundary thickness of 1 mm proving their capacity to improve US imaging in these tumors. Rattle-type MSN have exhibited higher US contrast than MSN or hollow MSN because of the existence of two interfaces that generate double scattering/reflection in comparison with the other types of nanoparticles that present only one interface [55]. The incorporation of enzyme-degradable shells on MSN as polycaprolactone has been reported to accelerate the nanoparticle degradation in biological fluids which contains lipases. This strategy improves the nanocarrier biocompatibility at the same time that increases its elasticity and US contrast agent behavior [56]. US have also been used to trigger the drug departure from MSN. Paris et al. have coated the MSN surface with a US-sensitive polymeric shell in order to retain cytotoxic drugs within the silica matrix until US exposition [57]. The stimuli-responsive release is provided by the incorporation of the thermosensitive polymer poly (2-(2-methoxyethoxy) ethyl methacrylate) (p (MEO_2_MA)) which contains 2-tetrahydropyranyl methacrylate (THPMA). This type of thermosensitive polymers exhibits linear-to-globular transitions in response to temperature rise and this transition temperature can be tuned by the introduction of hydrophilic monomers into the polymer composition. Under 10 min of US irradiation, the acetal group of THPMA was cleaved forming hydrophilic methacrylic acid (MAA) which increased the transition temperature of the polymer opening the pores that triggered the drug release (Figure 2).

One of the main limitations of the use of nanoparticles as drug carriers is their poor penetration in the tumoral tissue. Nanoparticles can reach the tumoral zone by EPR effect but once there, their penetration into the tissue is hardly compromised as a consequence of the presence of positive intratumoral pressure which pushes the nanocarriers away from the tumor [58] and by the slow diffusion rate of nanometric objects [59]. These limitations can be partially overcome by the incorporation proteolytic enzymes into the nanocarrier surface to digest the extracellular matrix and enhance the diffusion of the nanocarrier within the cancerous tissue [60]. One interesting approach to improve nanoparticle penetration is the use of focalized US to propel them into the tissue. The co-injection of dye-loaded MSN with sub-micrometric cavitation nuclei that enhances the US effect produced a significantly increased penetration of MSN in in vitro tumoral blood vessel models by the application of US [61]. MSN were extravasated from the blood vessel propelled by the violent collapse of gas bubbles generated by inertial cavitation. The co-administration of cavitation nuclei reduced the pressure required to induce inertial cavitation by half which allows to propel the nanocarriers employing conventional clinical US apparatus.

## 5. Magnetic Resonance Theranostics

**Figure 3:**
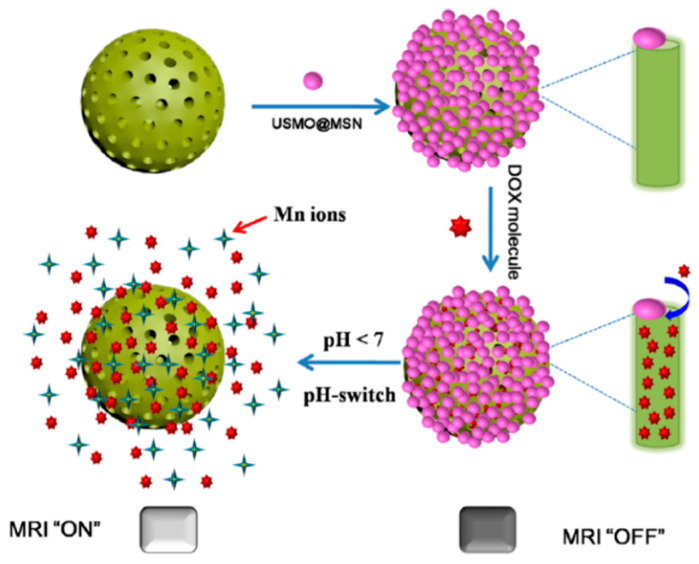
Mechanism of drug release and MRI contrast enhancement of MSN@USMO by dissolution of manganese nanoparticles in mild acidic conditions. Reproduced with permission from [65], American Chemical Society, 2018.

The application of magnetic fields to interrogate the disease progression by magnetic resonance imaging (MRI) has important advantages such as excellent tissue penetration, high spatial resolution, and harmless radiation. There are two main types of contrast agents for MRI: gadolinium (Gd) or manganese chelates [62], as T_1_-weighted positive agents and superparamagnetic iron oxide nanoparticles (SPION) [63], as T_2_-weighted negative agents, respectively. MRI is based on the relaxation of proton spin in the presence of a strong magnetic field. When a magnetic field is present, proton spin can be aligned parallel or antiparallel to the magnetic field direction. By the application of specific radiofrequencies (RF), it is possible to excite the proton spin to antiparallel orientation which will recover its low-energy state (parallel) when RF is ceased by two different relaxation mechanisms: longitudinal or T_1_ relaxation and transverse or T_2_ relaxation [63]. The use of these contrast agents enhances the variations between healthy and diseased tissues. Gd complexed with bovine serum albumin have been anchored on Dox-loaded MSN to act as redox-responsive poreblockers and MRI contrast agent [64]. The selectivity of this system against breast cancer cells was enhanced by grafting hyaluronic acid (HA) on MSN because of its high binding efficacy to the CD44 receptor located on the membrane of these tumoral cells. Thus, once the nanocarrier are engulfed by the tumoral cell, the higher glutathione concentration in the cytosol triggers the Dox release while the presence of Gd allows the in vivo monitorization of the nanocarrier biodistribution by MRI. Ultrasmall manganese oxide nanoparticles (USMO) (around 3 nm of diameter) were synthesized on the pore outlets of MSN forming core-shell nanoparticles MSN@USMO able to retain Dox in the pore network [65]. Drug release takes place by dissolution of the manganese nanoparticles which is accelerated in the mild acidic conditions present in the tumoral environment. Interestingly, the release of Mn^2+^ which happened in the USMO dissolution process enhanced the T1-weighted MR contrast capacity providing excellent MRI images of the diseased zone (Figure 3).

In other example, MSN loaded with tirapazamide, a bioreductive prodrug, which is only toxic in hypoxic cells, was alternatively coated with per-O-methyl-b-cyclodextrin-grafted-hyaluronic acid (HA-CD) and the porphyrin photosensitizer TPPS4 via layer-by-layer (LbL) assembly strategy [66]. Gd^3+^ was introduced in the system by chelation with TPPS4. This system was selectively engulfed by squamous cell carcinoma tumoral cells which overexpress CD44 receptor that binds to HA and release their payload in the presence of hyaluronidase, which is usually present in tumoral tissues. This nanocarrier is capable of destroying the tumoral cells by the synergic action of the released tirapazamide and singlet oxygen species produced by NIR laser radiation owing to the presence of photosensitizers. Additionally, the nanocarrier biodistribution can be monitored by fluorescence imaging and MRI.

SPION combine their ability to act as T_2_-relaxation contrast agent with their capacity to increase the surrounding temperature in the presence of an alternative magnetic field (AMF). This last property has been widely employed to destroy solid tumors by magnetic hyperthermia because tumoral cells are more sensitive to temperature than healthy ones [67]. SPION have been incorporated in MSN (MSN@SPION) to provide magnetic-responsive properties which have been used to trigger the release of different therapeutic agents [68]. This approach presents the advantage of the synergism achieved with the temperature increase in the tumoral tissue that enhances the effect of some chemotherapeutic agents [69]. Cai et al. have developed MSN which contains SPION to transport Dox to breast cancer cells following their accumulation in murine models by MRI [70]. In other work, Shi et al. have anchored SPION on amine-functionalized hollow MSN surface exploiting the affinity of SPION surface by amino groups [71]. The surface of these nanocarriers was coated with polydopamine which improved their colloidal stability and served as NIR absorber that allowed the destruction of tumoral cells by photothermal therapy. The accumulation of nanoparticles on the tumoral tissue was followed by MRI and photoacoustic imaging. MSN@SPION have been employed in suicide gene therapy for hepatocellular carcinoma [72]. This therapy is based on the introduction of genes into the tumoral cell genome to express specific enzymes that activate pro-drugs provoking the cellular destruction. In this work, the herpes simplex virus thymidine kinase/ganciclovir (HSV-TK/GCV) is conjugated with a copolymer of PEG and poly-lysine which provides positive charges on the plasmid conjugate that allows the retention on the surface of negatively charged MSN surface, which was previously functionalized with carboxylate groups. The adsorption of the plasmid on the nanocarrier surface increases its protection against external insults and enhanced its blood circulation time. Moreover, the presence of poly-lysine facilitated the endosomal escape of the nanocarrier once engulfed by tumoral cells by proton-sponge effect. The application of an alternative magnetic field provoked the temperature to increase up to 46 °C which enhanced the effect of suicide gene therapy because hyperthermia roused the dormant cells in G0 phase making them more susceptible to gene transfection. Finally, the use of external magnetic fields improved the retention of these nanocarriers in the tumoral tissue which was monitored by MRI. MSN@SPION decorated with folic acid as targeting agent have been employed to selectively deliver quercetin, a natural flavonoid with multiple antitumoral properties, to human colorectal carcinoma cells [73]. Instead of SPION, other magnetic alloys in nanoparticulate state that FePt is incorporated in MSN to provide magnetic hyperthermia and MRI contrast agent capacities [74]. One important advantage is that FePt nanoparticles exhibit NIR absorption that allowed to combine the capacity to release chemotherapeutic drugs located within the pore network of MSN with PTT.

## 6. Radionuclide-Based Theranostics

**Figure 4:**
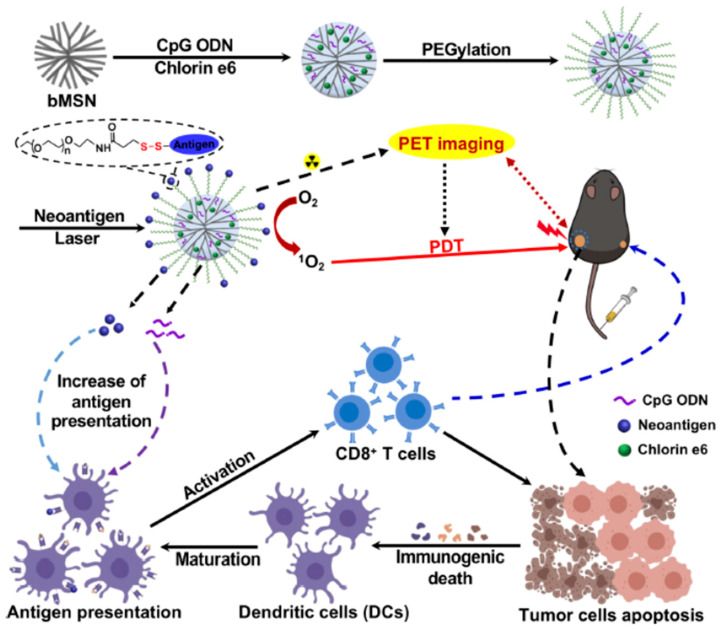
MSN able to induce antitumoral response by synergic effect of the release of immunostimulating agents CpG and neoantigens and PDT. The animals exhibited a significant tumor reduction even in tumors located in non-irradiated flank confirming the abscopal antitumoral effect. Reproduced with permission from [77], American Chemical Society, 2019.

The incorporation of radioactive elements in nanoparticles allows their detection employing different techniques being positron emission tomography (PET) one of the most widely used. PET is based on the emission of positrons by radioactive elements which are combined with electrons of the surrounding tissue producing gamma-rays that are detected producing 3D imaging of the tissue. ^18^F is a popular positron emitter which is transported to the surface of MSN for tumor imaging by PET following a two-step process based on biorthogonal click chemistry reactions [75]. In this elegant strategy, MSN were functionalized with a strained cyclooctine, dibenzocyclooctyne (DBCO) and the nanoparticles were injected in the tail of tumor-bearing mice. After 24 h, the nanoparticles were accumulated in the tumoral tissue by EPR effect, azide-PEG-^18^F was injected being retained in the tumoral tissue because of the specific reaction between the azide groups and DBCO allowing the tumor imaging by PET. A similar recognition process has been reported employing ^64^Cu-tetrazine molecular PET probe [76]. In this work, MSN were employed to deliver a polymer which contained trans-cyclooctine to the tumoral tissue. The released macromolecules reacted with the PET probes which were administered separately by the specific reaction between the tetrazine and cyclooctyne group of each moiety retaining the ^64^Cu in the tumoral tissue. Large-pore MSN that contained neoantigen peptides, specific immunostimulating oligonucleotide strands (CpG), ^64^Cu, and chlorin e6 have been employed to trigger immune responses against tumors by combination of PDT, because of the presence of chlorin e6, and the release of immunostimulating agents [77]. These nanocarriers were administered in bilateral two-tumor model with MC-38 colon carcinoma. Once confirmed their accumulation in the tumoral lesions by PET, tumors located in the right flank of the animals were irradiated with NIR laser (660 nm, 50 mW/cm^2^ during 15 min) while the left-flank tumors were not irradiated (Figure 4). The animals treated with these nanocarriers showed a significant tumor shrinkage even in the non-irradiated tumors by abscopal effect that proves the efficient recruitment of immune system in the antitumoral response. The radical oxidative species released by PDT provoked the immunogenic cell death of the tumoral cells, which is a specific death mechanism that release danger signals to the surroundings that stimulated immune responses [78]. PDT in combination with the release of immunostimulating agents induced dendritic cell stimulation and CD8 + T cell eliciting effective antitumoral immune response.

Hollow MSN have been coated with perylene diimide forming hollow mesoporous organosilica (HMPDIN) which exhibited enhanced fluorescence and PA imaging capacity [79]. Additionally, ^64^Cu was incorporated on the surface by chelation with thiol groups previously introduced on HMPDIN surface. Finally, a hydrophobic antitumoral drug derived from camptothecin (SN38) was loaded. The biodistribution in glioblastoma bearing mice models of these nanoparticles was followed by PA and PET imaging while 671 nm laser irradiation of tumoral lesions induced drug departure and hyperthermia provoking a significant tumor reduction and prolonged survival rate. Other radionuclides as ^89^Zr [80] and ^99^Tc [81] have been loaded on MSN surface to allow their visualization by PET.

## 7. Conclusions

The use of nanoparticles as drug carriers provides an excellent opportunity to deliver therapeutics to diseased tissues with high selectivity and fine control in the drug release process. These properties have paramount importance in the treatment of solid tumors because of the high toxicity of the conventional antitumoral drugs. The passive accumulation of nanocarriers in tumoral tissues by EPR in combination with the nanocarrier surface decoration with targeting moieties reduces the side toxicity associated to antitumoral drugs. Theranostic nanocarriers provide one additional property which is the possibility to monitor their localization into the host and to provide information about disease progression and the effect of their own therapy. Therefore, multifunctional nanocarriers endowed with imaging and controlled release properties have been described to treat malignancies yielding information about their efficacy simultaneously. Among the different types of nanocarriers MSN constitute an excellent platform because of their unique properties. The external surface of MSN can be easily functionalized with multiple chemical groups allowing to anchor a vast number of imaging agents, from small fluorophores to other nanoparticles able to act as contrast agents in multiple imaging techniques. This property, in combination with the excellent cargo capacity of this material convert MSN into a powerful theranostic nanomaterial (Table 1). Silica is considered as generally recognized as safe (GRAS) material by US Food and Drug Administration (FDA) and several studies have concluded that MSN are biocompatible and harmless [82]. However, knowledge is needed in order to evaluate the safety of MSN depending of the administration route, particle size, surface decoration, and degradability in physiological fluids [83,84]. Additionally, long-term toxicity studies about the chronic administration of MSN are needed. Recently, Mohammadpour et al. have evaluated the effect of the intravenous administration of silica nanoparticles with different sizes and porosities for one year in animal models showing scarce toxicity [85]. The development of industrial synthetic routes which yield large amounts of high quality MSN with reproducibility between batches is also a challenge. There is much work to do but the future of these MSN theranostics is full of potential and they would provide powerful tools in the oncology clinical field.

## Figures and Tables

**Table 1:** Summary of the different theranostics MSN according with the imaging technique, payload, and stimuli which trigger the therapeutic response.

Type	Stimuli	Payload	Therapeutic Application	Ref
Optical	NIR	Indocyanine green (ICG)	PDT	[38]
	MMP	PLGVR peptide and CPT	Chemotherapy	[39]
	NIR	DIR	PTT	[40]
	NIR	Aptamer YQ26 and Cy5.5	Fluorescent imaging of tumoral blood vessels	[41]
	Gluthation/caspases	Polydisulfides, small molecule inhibitors, anti-miR-21	Chemotherapy	[42]
	VIS	CD and Dox	Chemotherapy	[43]
	NIR	CuS	Hyperthermia	[46]
Photoacustical	NIR	ICG and MTX	Chemotherapy	[47]
	NIR	ICG and NaYF4/Yb/Er nanoparticles	Hyperthermia	[48]
	NIR	Gd and Dox	Hyperthermia/chemotherapy	[49]
	NIR	PFP and gold nanorods	Hyperthermia	[50]
Ultrasound	US	p(MEO2MA)/THPMA and Dox	Chemotherapy	[57]
	US	Co-injection with cavitation nuclei	Tumoral tissue penetration enhancers	[61]
Magnetic resonance	Glutathione	Gd@BSA/Dox	Chemotherapy	[64]
	pH	USMO/Dox	Chemotherapy	[65]
	Hyaluronidase/NIR	HA-CD/Gd/tirapazamide	Chemotherapy/PDT	[66]
	Magnetic field	PNIPAM/NHMA/SPION/Dox	Chemotherapy/hyperthermia	[69]
	NIR	Polydopamine/SPION	Hyperthermia	[71]
	Magnetic field	Polylysine/HSV-TK/GCV/SPION	Gene suicide/Hyperthermia	[72]
Radionucleotide-based	NIR	neoantigen peptides/CpG/chlorin e6	Immunotherapy/PDT	[77]
	NIR	^64^Cu/SN38	Chemotherapy	[79]

## Abbreviations List

CD	carbon dots
CPT	camptothecin
DBCO	dibenzocyclooctyne
Dox	doxorubicin
ECM	extracellular matrix
EPR	enhanced permeation and retention effect
FDA	food and drug administration
HER2	human epidermal growth factor receptor 2
ICG	indocyanine green
MRI	magnetic resonance imaging
MSN	mesoporous silica nanoparticles
MTX	mitoxantrone
NIR	near infrared radiation
PA	photoacoustic imaging
PDT	photodynamic therapy (PDT)
PEG	polyethyleneglycol
PET	positron emission tomography
PFP	perfluoropentane
PTT	photothermal therapy
RF	radiofrequencies
SPION	superparamagnetic iron oxide nanoparticles
THPMA	2-tetrahydropyranyl methacrylate
US	ultrasounds
UV	ultraviolet light
VIS	visible light
